# Relationship between ear appearance and psychological function among congenital unilateral microtia after autologous cartilage ear reconstruction: a chain mediation model

**DOI:** 10.1186/s40359-025-03073-5

**Published:** 2025-07-24

**Authors:** Xinyu Li, Chenlong Li, Tianyu Zhang, Jing Xu, Yaoyao Fu

**Affiliations:** 1 Department of Facial Plastic and Reconstructive Surgery, Eye & ENT Hospital, Fudan University, Shanghai, China; 2 ENT Institute, Eye & ENT Hospital, Fudan University, Shanghai, China; 3 NHC Key Laboratory of Hearing Medicine, Fudan University, Shanghai, China; 4 Department of Nursing, Eye & ENT Hospital, Fudan University, Shanghai, China

**Keywords:** Congenital unilateral microtia, Ear appearance, Psychological function, Social identity theory, Chain mediation model

## Abstract

**Background:**

Congenital microtia significantly impacts children’s psychological health, yet there has been limited focus on the effect of ear reconstruction surgery on psychological improvements, especially its pathway. This study aims to explore the role of ear appearance in improving psychological health in individuals with congenital unilateral microtia based on social identity theory, highlighting the limited understanding of the specific pathways involved in this relationship, and investigating how social function and benefit mediate the connection between ear appearance and psychological health in patients who underwent autologous cartilage ear reconstruction.

**Methods:**

A cross-sectional study was conducted, involving 96 patients with congenital unilateral microtia between January and June 2024 at the Eye & ENT Hospital of Fudan University. Sociodemographic and clinical data were collected, along with responses to the EAR-Q and Glasgow Children’s Benefit Inventory (GCBI) questionnaires. Data analysis was performed using multiple linear regression and the PROCESS macro in SPSS.

**Results:**

Mediation analysis revealed that social function and emotion mediated the relationship between ear appearance and psychological function (total effect = 0.82; direct effect = 0.21; indirect effect = 0.61). Three mediation pathways were identified: ear appearance influenced psychological function through social function, emotional benefit, and a combined effect of both.

**Conclusions:**

These findings underscore the critical role of social interaction and emotional health in shaping psychological outcomes for individuals with congenital unilateral microtia following reconstruction. The results offer valuable insights for developing targeted interventions to enhance psychological well-being in this population.

## Introduction

Microtia, a congenital condition marked by the underdevelopment of the external ear, affects approximately 3.6 per 10,000 live births in China [1]. Unilateral microtia, which accounts for about 70% of cases, often leads to difficulties in sound localization and speech recognition, particularly in noisy environments. These auditory challenges can hinder language development and academic performance [2]. The ear is essential for facial symmetry and balance, contributing to overall facial harmony [3]. The facial asymmetry caused by microtia can negatively affect body image, a concept that begins to form in early childhood through comparison with others and becomes internalized over time [4]. This altered body image can profoundly impact self-concept, leading to long-term effects on psychological health and overall well-being [5]. Consequently, children with unilateral microtia are particularly vulnerable to low self-esteem [6] and body image disorders [7].

Ear reconstruction surgery has been shown to significantly improve patients’ mental health, but the underlying pathways remain unclear. Techniques such as autologous ear reconstruction and porous polyethylene ear reconstruction have been proven to enhance ear aesthetics significantly [8, 9]. Ronde and colleagues found that ear reconstructive surgery for microtia notably improves psychological well-being, reducing negative emotions and enhancing social functioning [10]. While much research has focused on the technical aspects of ear reconstruction and auditory rehabilitation [11], the psychological benefits, including increased self-confidence and social acceptance, are equally important [10, 12]. As a result, individuals who undergo ear reconstruction often experience better social adjustment, leading to improved quality of life, health-related benefits, and greater personal satisfaction [13]. However, the exact pathways through which ear reconstructive surgery impacts psychological health in individuals with microtia remain unclear. Understanding how these mental health improvements occur is crucial for achieving a truly comprehensive recovery and enhancing overall well-being.

**Fig. 1:**
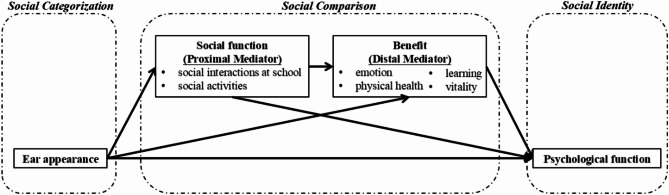
Hypothesized model of mediations

To address this gap, our study seeks to explore the pathway between ear appearance and psychological health through the lens of social identity theory (SIT). Social identity, which refers to an individual’s sense of belonging to a group, is shaped by the categorization of certain characteristics [14]. The key psychological processes of social identity include social categorization, social comparison, and the formation of social identity [15]. For children with visible physical deformities like microtia, the sense of belonging to a different group can make social interactions particularly challenging. These children often face adverse childhood experiences that differ significantly from those of their peers [16, 17]. Many children with microtia experience social stigma, discrimination, and exclusion, which can hinder their ability to form meaningful relationships [18], leading them to feel increasingly disconnected in social settings. This shift in social identity, as part of the broader self-concept, can be influenced by negative social feedback, such as social anxiety [12, 19], which in turn exacerbates mental health challenges and related physical and psychological functions. In the context of ear reconstruction surgery, its benefits must also be considered [10]. Although there is evidence that these surgeries improve psychosocial well-being, the underlying relationships between these benefits and other health-related factors remain underexplored. To investigate these connections, we propose the hypothesized model shown in Fig. 1.

The primary objective of this study is to investigate the relationships among social function, perceived benefits, ear appearance, and psychological function in individuals with congenital unilateral microtia based on SIT. A secondary aim is to examine the mediating roles of social function and benefit in these relationships. We hypothesize that: (1) ear appearance is positively associated with social function, benefit, and psychological function; (2) social function and benefit are positively associated with psychological function; and (3) social function mediates the relationship between ear appearance and psychological function through the influence of benefit.

## Materials and methods

### Study design, setting, participants and ethics

This study employed a cross-sectional design, conducted at the Eye & ENT Hospital of Fudan University, Shanghai, China, between January and June 2024. Informed consent was obtained from all participants. For individuals under 16 years of age, consent was provided by a parent/legal guardian and child. Participants aged 16 and older provided their own consent. The study received ethical approval from the Ethics Committee and the Ethics Review Board of Eye & ENT Hospital of Fudan University (No. 2023191). A Monte Carlo power analysis for indirect effects [20], using the correlation matrix from the pre-trial data, determined that a sample of 88 participants was required to replicate the chain mediation findings with 80% power.

### Inclusion and exclusion criteria

The inclusion criteria for the study were as follows: (a) participants aged between 8 and 18 years, (b) diagnosis of congenital unilateral microtia, and (c) presence of hearing impairment. Exclusion criteria included: (a) severe systemic illnesses, such as heart, liver, lung, or kidney dysfunction, (b) mental abnormalities or cognitive impairment, (c) acquired ear deformity, and (d) craniomaxillofacial deformities.

### Study procedures

The researcher provided comprehensive information about the trial to potential participants and collected baseline assessments, which encompassed demographic, household, and clinical data, following the acquisition of informed consent. The surgical approach employed in this study was based on the technique described by Nagata [21]. Stage I of the procedure involved the implantation of a cartilage framework, lobule transposition, and tragus construction. Approximately six months later, Stage II consisted of ear elevation. All participants were recruited, and questionnaires were administered prior to their secondary admission for Stage II of the reconstruction surgery.

### Measures

#### Demographic and clinical data

Parents or guardians completed a brief self-designed questionnaire providing demographic and household information, including the child’s age, gender, place of residence, and household wealth. Additionally, medical records, including healthcare history, routine physical examinations, and pure tone audiometry reports, were obtained from outpatient records.

#### Ear questionnaire (EAR-Q) scales

The EAR-Q is divided into three Sections [22]. For this study, we focused on the “Appearance” and “Health-Related Quality of Life (HRQoL)” sections. The “Appearance” section assesses participants’ satisfaction with the appearance of their ears. The HRQoL section evaluates psychological, school, and social aspects. Specifically, the psychological scale examines self-esteem, body image, and confidence; the school scale assesses social interactions at school, such as making friends and enjoying school; and the social scale measures social activities, acceptance by peers, fitting in, and feeling valued. Both the school and social scales evaluate social function in this study. Each scale consists of 10 positively phrased items, rated on a 4-point Likert scale. The “Appearance” section reflects current feelings, while the HRQoL section pertains to experiences over the past week. Total raw scores are computed for each scale and converted to a 0 to 100 scale using Rasch analysis, with higher scores indicating better outcomes [23]. The Cronbach’s α for EAR-Q scale was 0.94 [24]; in this study the Cronbach’s α was 0.87.

#### Glasgow children’s benefit inventory (GCBI) questionnaire

GCBI is a validated tool used to evaluate health-related benefits in pediatric patients following otolaryngological treatments [25]. The GCBI comprises 24 questions across four domains: emotion, physical health, learning, and vitality. Each item is rated on a 5-point Likert scale, where the midpoint indicates “no change,” and the endpoints represent “much better” and “much worse.” The final score is calculated by summing all item scores, dividing by the number of items, and multiplying by 50. This results in a score ranging from − 100 to + 100, with negative scores reflecting a deterioration in quality of life and positive scores indicating an improvement. The Cronbach’s α for this questionnaire was 0.92 [25]; in this study the Cronbach’s α was 0.73.

### Data analysis

Quantitative data were presented as means ± standard deviations (SD), while categorical data were described using frequencies and percentages. Missing values were corrected using a mean value imputation. A paired sample t-test was employed to compare EAR-Q scores before and after surgery. To identify statistically significant differences between patient characteristics and psychological function, one-way ANOVA was used for univariate analysis. Pearson correlation analysis was performed to examine relationships between variables. Variables that showed significant differences in the univariate analysis were then included in stepwise multiple linear regression models. The variance inflation factor (VIF) was calculated for each independent variable to assess multicollinearity. To explore the relationship between ear appearance and psychological function, with social function as proximal mediator and benefit as distal mediator, we used the PROCESS model 6 [26]. In the final chain mediation model, ear appearance was designated as the independent variable (X), with psychological function as the outcome variable (Y). HRQoL-Social, representing social activities, served as the proximal mediator (M1), while emotion from the benefit sub-domain was treated as the distal mediator (M2); these variables were assessed using the EAR-Q for ear appearance, psychological function, and HRQoL-Social, and the GCBI for emotion. Effect values for each pathway were reported. Statistical significance for direct, indirect, and total effects was determined using 5,000 bootstrap resamples and examining the confidence intervals (CIs) to see if they included zero. Since we adjusted the variables in the hypothesized mediation model during the mediation effect analysis, a Monte Carlo-based post-hoc power analysis was conducted to confirm whether 80% power was achieved to reliably estimate each parameter in the final model.

## Results

### Participants’ characteristics & baseline analyses

118 eligible patients were approached, of which 102 consented to participate (86.4%). After eliminating 6 invalid questionnaires, there are 96 valid questionnaires. Table 1 presents the characteristics of the participants. The cohort was predominantly male (69.79%) with a mean age of 11.01 ± 2.26 years. Most participants had undergone reconstructive surgery on the right ear (65.62%), were in primary school (88.54%), came from nuclear families (73.96%), and were primarily cared for by their mothers (62.50%). None of the participants had any chronic illnesses. Table 2 shows that all four GCBI domains—emotion, physical health, learning, and vitality—as well as the overall benefit score, were positive, indicating that participants perceived meaningful postoperative benefits following ear reconstruction. Table 3 displays the results of the EAR-Q scales before and after surgery. Statistically significant improvements were observed in appearance-ear, HRQoL-psychological, HRQoL-school, and HRQoL-social following surgery (*p* < 0.05). Table 4 summarizes the univariate analysis of HRQoL-psychological, highlighting significant differences related to only-child status (*p* = 0.01) and medical payment (*p* < 0.01).

### Bivariate correlations among all the variables

**Fig. 2:**
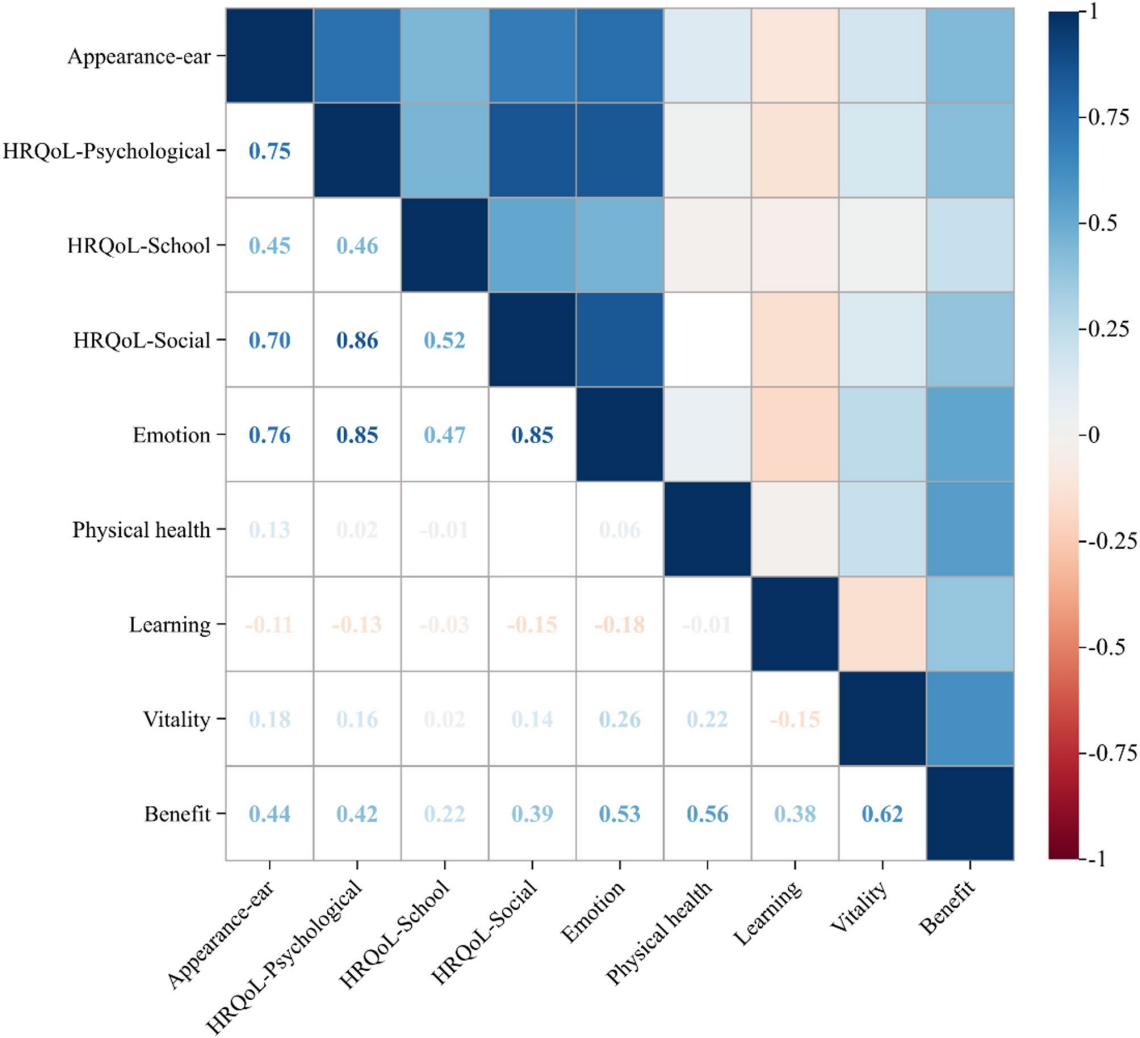
Heatmap of correlation analysis

Figure 2 illustrates the positive correlations between HRQoL-psychological, appearance-ear, and other variables. HRQoL-psychological showed significant positive correlations with appearance-ear (*r* = 0.75, *p* < 0.01), HRQoL-school (*r* = 0.46, *p* < 0.01), HRQoL-social (*r* = 0.86, *p* < 0.01), emotion (*r* = 0.85, *p* < 0.01), and benefit (*r* = 0.42, *p* < 0.01). Appearance-ear was positively correlated with HRQoL-school (*r* = 0.45, *p* < 0.01), HRQoL-social (*r* = 0.70, *p* < 0.01), emotion (*r* = 0.76, *p* < 0.01), and benefit (*r* = 0.44, *p* < 0.01). HRQoL-social was positively associated with HRQoL-school (*r* = 0.52, *p* < 0.01), emotion (*r* = 0.85, *p* < 0.01), and benefit (*r* = 0.39, *p* < 0.01). Additionally, HRQoL-school and emotion were positively correlated (*r* = 0.47, *p* < 0.01).

### Multiple linear regression analysis of psychological function

Independent variables selected for the multiple linear regression analysis included only-child status, medical payment, HRQoL-school, HRQoL-social, the four domains of the GCBI (emotion, physical health, learning, and vitality), and appearance-ear. The stepwise multiple linear regression analysis revealed that HRQoL-social (β = 0.46, *p* < 0.01), emotion (β = 0.31, *p* < 0.01) and appearance-ear (β = 0.19, *p* = 0.01) were positively associated with HRQoL-psychological (Table 5). The model showed a reasonable fit with an R^2^ of 0.81 and an adjusted R^2^ of 0.80. Since the VIF for all independent factors ranged from 2.39 to 4.42, it indicates no significant collinearity.

### Chain mediation effect analysis

**Fig. 3:**
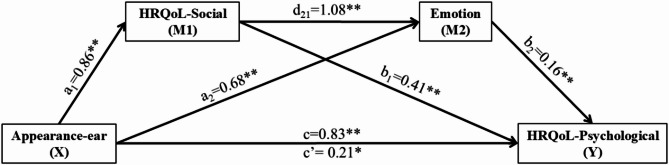
Mediation model: Ear appearance on psychology mediated by society and emotion

To explore the mediation effects, we employed SPSS macro PROCESS with 5000 bootstrap samples to test the mediation model 6. The results of the mediation analysis are summarized in Tables 6, and the unstandardized regression coefficients for the parallel mediation model are illustrated in Fig. 3. In this model, appearance-ear was treated as the independent variable (X), HRQoL-psychological as the dependent variable (Y), with HRQoL-social (M1) and emotion (M2) serving as mediating variables. The standardized regression coefficients (*β*) presented in Table 6 have been labeled and mapped onto the corresponding paths in Fig. 3, as detailed below. The regression analysis revealed that appearance-ear significantly and positively predicted HRQoL-psychological (c). Additionally, appearance-ear was a significant positive predictor of both HRQoL-social (a_1_) and emotion (a_2_). HRQoL-social positively predicted both emotion (d_21_) and HRQoL-psychological (b_1_). Moreover, appearance-ear (c’) and emotion (b_2_) were significant positive predictors of HRQoL-psychological.

Table 7 indicates a significant mediating effect of both HRQoL-social and emotion on the relationship between appearance-ear and HRQoL-psychological. The total indirect effect was 0.61 (SE = 0.17, bootstrap 95% CI: [0.33, 0.95]), representing 74.39% of the total effect (0.82). The direct effect of appearance-ear on HRQoL-psychological (c’) was also significant (direct effect = 0.21, SE = 0.08, bootstrap 95% CI: [0.06, 0.37]), suggesting that HRQoL-social and emotion partially mediate the effect of appearance-ear on HRQoL-psychological. The mediation analysis identified three distinct pathways: (1) the independent mediating effect of HRQoL-social (a_1_*b_1_), which accounted for 42.68% of the total effect (mediating effect = 0.35, SE = 0.22, bootstrap 95% CI: [0.05, 0.76]), (2) the independent mediating effect of emotion (a_2_*b_2_), which accounted for 13.42% of the total effect (mediating effect = 0.11, SE = 0.04, bootstrap 95% CI: [0.04, 0.21]), and (3) the chain mediating effect of HRQoL-social and emotion (a_1_*d_21_*b_2_), which accounted for 18.29% of the total effect (mediating effect = 0.15, SE = 0.07, bootstrap 95% CI: [0.06, 0.34]). The post-hoc Monte Carlo power analysis (*N* = 96; 10,000 replications; 95% CI) demonstrated adequate power for the model (1-*β*_a1*b1_ = 1, 1-*β*_a2*b2_ = 0.89, 1-*β*_a1*d21*b2_ = 0.89).

## Discussion

Microtia is relatively uncommon, and the sample for this study was drawn from the largest ear reconstruction center in China, known for its extensive microtia population. Consequently, the sample size is both large and representative, enhancing the generalizability of the results. Our study employed robust statistical analyses to explore the impact of ear appearance on psychological function in children who have undergone unilateral reconstructive surgery for microtia based on SIT. The findings revealed significant improvements in psychological, school, and social functions post-surgery, underscoring the substantial positive effects of ear reconstruction on HRQoL. The chain mediation analysis further elucidated that social function and emotion mediate the relationship between ear appearance and psychological function, highlighting the interconnected nature of physical and psychological health. The substantial total indirect effect, accounting for 74.39% of the overall effect, underscores the significant role of social function, which contributed 42.68% of the total effect. This result aligns with existing literature that emphasizes the positive impact of improved physical appearance on psychosocial well-being [12] and corroborates previous research indicating that improvements in psychological function are largely mediated by social interactions and emotional well-being [2, 13, 16]. Children with microtia frequently face social challenges such as teasing, bullying, and exclusion [10, 27], making the role of social function particularly crucial [28]. These challenges are deeply intertwined with the children’s social identity, as their sense of belonging and self-worth is affected by how they perceive themselves in relation to others. SIT posits that individuals derive a significant part of their self-concept from group membership, and for children with visible deformities like microtia, the stigma they face often leads to a sense of exclusion and diminished social identity [29]. The improvements in social function observed in our study can be seen as a reflection of the enhanced social belonging that follows from ear reconstruction, as the physical change in appearance allows these children to feel more aligned with their peers and accepted in social contexts.

Additionally, our findings highlight that emotion, as an outcome of otolaryngological surgery, independently contributes to psychological function, emphasizing the need to address emotional well-being in these children. According to SIT, emotional responses such as pride or satisfaction with one’s appearance can reinforce a positive social identity, thereby improving psychological outcomes [30]. Negative emotions, conversely, can further entrench feelings of social exclusion and contribute to a negative self-concept. Childhood emotional issues, if left unaddressed, can lead to long-term psychological and behavioral problems [31]. Steffen’s research supports our findings, demonstrating that improvements in psychological well-being following auricular reconstruction are closely linked to positive emotional acceptance and satisfaction with the reconstructed ear [32]. Furthermore, the direct effect of ear appearance on psychological function indicates that, although social function and emotion are critical, ear appearance itself also independently enhances psychological outcomes. This finding supports the idea that ear reconstruction not only enhances social integration but also improves self-esteem and body image, leading to better psychological health. This improvement in self-esteem is indicative of a shift in the child’s social identity towards a more positive and integrated sense of self [33]. By reducing the physical stigmas that contribute to a negative social identity, ear reconstruction surgery fosters a more positive self-concept, facilitating improved psychological and emotional health [10, 12].

In this study, we employed the EAR-Q and GCBI tools to comprehensively assess HRQoL and patient-reported outcomes in children with ear conditions, with a focus on the psychological impact of ear reconstruction. The EAR-Q specifically measures patient-reported outcomes related to congenital ear abnormalities [10, 12], while the GCBI evaluates benefits resulting from otolaryngological surgery [25]. Postoperative assessments revealed positive improvements in overall scores and across individual dimensions, indicating significant benefits for patients. The significant increase in postoperative EAR-Q scores compared to preoperative levels reflects the success of ear reconstruction surgery in enhancing multiple aspects of patients’ well-being. These results are consistent with the findings of Johns [34], which highlighted the positive impact of ear reconstruction on both social and psychological functions. Moreover, the benefits of ear reconstruction extend beyond physical appearance. By addressing visible differences, the surgery mitigates the risk of discrimination and its associated negative psychological effects on the child. This underscores the multifaceted advantages of ear reconstruction in improving both physical and psychosocial outcomes.

In this study, we employed univariate analysis, correlation analyses, and linear regression to systematically identify key factors influencing psychological function, ultimately focusing on social and emotional factors in the mediation analysis. The univariate analysis revealed that children from single-child families and those covered by health insurance demonstrated better psychological outcomes. This may be attributed to the reduced educational pressures in single-child households and the alleviation of financial burdens for insured families. Children with visible physical conditions often face considerable psychosocial challenges, underscoring the need for comprehensive, multidisciplinary support systems [35]. The exclusion of the benefit variable from the final model may be due to the weak association between most of its sub-variables and psychological function. Similarly, physical health improvements showed no significant correlation with other variables, reflecting the specific nature of ear reconstruction surgery. Variables such as school function, learning, and vitality also exhibited weak correlations, which may be explained by the extended school absences many patients experienced postoperatively, often lasting six months to a year. These prolonged absences likely reduced social interaction, exacerbating the psychological challenges faced by patients [36].

Another noteworthy point is that the auditory status of the patients is in accordance with previous research, showing poorer hearing on the affected side, with worse air conduction compared to bone conduction [37]. Asymmetric hearing loss can lead to “aural preference syndrome” [38], which significantly impacts auditory system development and speech, potentially affecting a child’s social engagement and personality development [39]. The combined effects of ear appearance and hearing loss likely contributed to poorer social and psychological outcomes prior to surgery. However, hearing outcomes were not included in the regression analysis, as patients who underwent auditory reconstruction were not specifically identified or excluded from this study. This may explain the limited improvements in body image concerns observed in some patients after surgery, similar to findings in cosmetic surgery research [40]. Future studies should further investigate the relationship between hearing impairment and psychological well-being in patients with microtia.

Our study examined the complex pathways connecting various factors related to psychological functioning in children with microtia, offering valuable insights for designing interventions to improve their psychological well-being. The self-concept of children with disabilities, particularly in relation to social identity, is heavily influenced by the inclusivity and supportiveness of their social environments, especially during social comparison [27]. This understanding highlights the importance of providing holistic postoperative care that goes beyond physical reconstruction, focusing also on fostering social capital and emotional support to strengthen psychosocial functioning in children and adolescents [6, 41]. Future research should explore the long-term psychological and social effects of reconstructive surgery, with a focus on key factors such as family support and peer relationships, in order to address the negative social identity often experienced by children with microtia. Investigating these factors will deepen our understanding of the sustained impact of ear reconstruction and inform the development of targeted interventions to support these children throughout their developmental stages [19, 42].

### Study limitations

This study has some limitations. First, the cross-sectional design limits the ability to infer causality between variables. Longitudinal studies are necessary to examine how changes in ear appearance influence psychological function over time. Second, the homogeneity of the sample, which predominantly includes participants from China. Future research should aim to include a more diverse population to validate these results across different demographic groups. Third, while mediation analysis provided insight into the pathways linking ear appearance and psychological health, the complexities of human psychology suggest that other unmeasured factors may also play a role. Further studies should explore additional mediators and moderators to deepen our understanding of these relationships.

**Table 1:** Participant demographics and clinical characteristics (*n* = 96)

Characteristics	Mean ± SD or *N* (%)
Age, years	11.01 ± 2.26
Weight, kg	39.29 ± 10.00
Height, m	1.45 ± 0.10
PTA, dB HL	
Constructed ear-AC	67.61 ± 11.36
Constructed ear-BC	21.38 ± 6.14
Normal ear-AC	14.85 ± 7.83
Normal ear-BC	7.71 ± 3.91
Operative side	
Right	63 (65.62)
Left	33 (34.38)
Gender	
Male	67 (69.79)
Female	29 (30.21)
Ethnicity	
Han	92 (95.80)
Others	4 (4.20)
Only child status	
Yes	69 (71.88)
No	27 (28.12)
Education level	
Primary school	85(88.54)
Middle school	7 (7.29)
High School	4 (4.17)
Family caregiver	
Mother	60 (62.50)
Father	35 (36.46)
Grandparents	1 (1.04)
Family type	
Nuclear family	71 (73.96)
Extended family	25 (26.04)
Family income, RMB	
3000–5000	12 (12.50)
5000–10,000	58 (60.42)
>10,000	26 (27.08)
Residence area	
Urban	81 (84.38)
Rural	15 (15.62)
Medical payments	
Medical insurance	75 (78.12)
Self-pay	21 (21.88)

Abbreviations: SD: standard deviation; PTA: pure tone audiometry; dB HL: decibels, hearing level; AC: air conduction, BC: bone conduction; HRQoL: health-related Quality of Life

**Table 2:** Benefit across GCBI domains and overall after surgery (*n* = 96)

Domain	Mean ± SD
Emotion	58.02 ± 23.69
Physical health	49.38 ± 30.15
Learning	45.49 ± 31.67
Vitality	63.31 ± 27.71
Total	54.46 ± 14.50

Abbreviations: SD: standard deviation; GCBI: Glasgow children’s benefit inventory

**Table 3:** Comparison of difference of EAR-Q after surgery (*n* = 96)

EAR-Q	Preoperative (Mean ± SD)	Postoperative (Mean ± SD)	t	*p*
Appearance-ear	33.27 ± 6.90	72.54 ± 11.09	-28.88	<0.01
HRQoL-Psychological	31.02 ± 7.55	72.15 ± 12.24	-28.99	<0.01
HRQoL-School	26.17 ± 6.47	71.00 ± 10.34	-39.2	<0.01
HRQoL-Social	42.73 ± 9.60	75.37 ± 13.71	-18.89	<0.01

**Table 4:** Characteristics of participants and univariate analysis of psychological health (*n* = 96)

Characteristics	HRQoL-Psychological (Mean ± SD)	F	*P*
Operative side		0.00	0.97
Right	72.19 ± 13.45		
Left	72.08 ± 9.72		
Gender		0.25	0.62
Male	72.56 ± 11.18		
Female	71.19 ± 14.57		
Ethnic		2.45	0.12
Han	72.55 ± 12.29		
Others	62.84 ± 6.17		
Only child status		7.87	0.01
Yes	74.27 ± 11.08		
No	66.74 ± 13.57		
Education level		0.74	0.48
Primary school	71.94 ± 12.49		
Middle school	70.63 ± 11.69		
High School	79.25 ± 5.32		
Family caregiver		3.10	0.05
Mother	73.06 ± 12.76		
Father	71.4 ± 10.48		
Grandparents	43.51 ± 0.00		
Family type		0.01	0.93
Nuclear family	72.09 ± 12.46		
Extended family	72.33 ± 11.82		
Family income, RMB		1.24	0.29
3000–5000	67.11 ± 15.27		
5000–10,000	72.54 ± 11.89		
>10,000	73.60 ± 11.38		
Residence area		0.51	0.48
Urban	72.54 ± 12.22		
Rural	70.07 ± 12.57		
Medical payments		12.99	<0.01
Medical insurance	74.39 ± 10.84		
Self-pay	64.13 ± 13.79		

**Table 5:** Multiple linear regression analysis (*n* = 96)

Variables	Unstandardized coefficients		Standardized coefficients		*P*	95% CI for β		Collinearity statistics
	B	SE	β	t		LL	UL	VIF
(Constant)	16.34	5.25		3.11	<0.01	5.92	26.77	
HRQoL-Social	0.41	0.08	0.46	5.23	<0.01	0.25	0.57	3.67
Emotion	0.16	0.05	0.31	3.26	<0.01	0.06	0.26	4.42
Appearance-ear	0.21	0.08	0.19	2.74	0.01	0.06	0.37	2.39

Notes: F = 128.16, *p*<0.01, R^2^ = 0.81, R^adj^ = 0.80

Abbreviations: SE: standard error; CI: confidence interval; LL: lower limit, UL: upper limit; VIF: variance inflation factor

## Conclusion

In our model of SIT, ear appearance plays a significant role in the psychological functioning of children following reconstructive surgery, with social function and emotional well-being serving as key mediators. These findings highlight the need for a holistic approach to care those addresses both the physical and psychosocial needs of children with microtia. Future research should further explore these relationships to gain a deeper understanding of the complexities surrounding psychological health in microtia patients, ultimately guiding the development of more effective intervention strategies.

**Table 6:** Mediation analysis (*n* = 96)

Outcome Variables	Covariates	*R*	*R* ^2^	F	β	SE	t	*p*
HRQoL-Social	Appearance-ear	0.70	0.58	88.07	0.86	0.09	9.38	<0.01
Emotion	Appearance-ear	0.88	0.77	159.02	0.68	0.15	4.65	<0.01
	HRQoL-Social				1.08	0.12	9.13	<0.01
HRQoL-Psychological	Appearance-ear	0.90	0.81	128.16	0.21	0.08	2.74	0.01
	HRQoL-Social				0.41	0.08	5.23	<0.01
	Emotion				0.16	0.05	3.26	<0.01
HRQoL-Psychological	Appearance-ear	0.75	0.56	120.91	0.83	0.08	11.00	<0.01

Abbreviations: SE: standard error

**Table 7:** Mediating effects of ear appearance on psychology

Model pathways	Effect	SE	t	*p*	Boot 95% CI		Effect sizes
					LL	UL	
Total effect	0.82	0.08	11.00	<0.01	0.68	0.98	100.00%
Direct effect	0.21	0.08	2.74	0.01	0.06	0.37	25.61%
Indirect effects	0.61	0.17			0.33	0.95	74.39%
Appearance-ear→ HRQoL-Social→ HRQoL-Psychological	0.35	0.22			0.05	0.76	42.68%
Appearance-ear→ Emotion→ HRQoL-Psychological	0.11	0.04			0.04	0.21	13.42%
Appearance-ear→ HRQoL-Social→ Emotion→ HRQoL-Psychological	0.15	0.07			0.06	0.34	18.29%

Abbreviations: SE: standard error; CI: confidence interval; LL: lower limit, UL: upper limit

## Data Availability

The datasets used or analyzed during the current study are available from the corresponding author on reasonable request.

## Abbreviations

CIs: Confidence intervals
ENT: Ears, nose, and throat
GCBI: Glasgow Children’s Benefit Inventory
HRQoL: Health-Related Quality of Life
SD: Standard deviation
SE: Standard error
SIT: Social Identity Theory
VIF: Variance inflation factor
